# Measuring quality of life in opioid-dependent people: a systematic review of assessment instruments

**DOI:** 10.1007/s11136-017-1674-6

**Published:** 2017-07-31

**Authors:** Lisa Strada, Wouter Vanderplasschen, Angela Buchholz, Bernd Schulte, Ashley E. Muller, Uwe Verthein, Jens Reimer

**Affiliations:** 1 Centre for Interdisciplinary Addiction Research, University Medical Centre Hamburg-Eppendorf, Martinistrasse 52, 20246 Hamburg, Germany; 2 Department of Special Needs Education, Ghent University, Henri Dunantlaan 2, 9000 Ghent, Belgium; 3 Department of Medical Psychology, University Medical Centre Hamburg-Eppendorf, Martinistrasse 52, 20246 Hamburg, Germany; 4 Norwegian Centre for Addiction Research, Institute of Clinical Medicine, University of Oslo, Pb 1039 Blindern, 0450 Oslo, Norway; 5 Gesundheit Nord, Kurfürstenallee 130, 28211 Bremen, Germany

**Keywords:** Quality of life, Opioid, Instrument, Review, Content analysis, Substance use disorder

## Abstract

**Purpose:**

Opioid dependence is a chronic relapsing disorder. Despite increasing research on quality of life (QOL) in people with opioid dependence, little attention has been paid to the instruments used. This systematic review examines the suitability of QOL instruments for use in opioid-dependent populations and the instruments’ quality.

**Methods:**

A systematic search was performed in the databases Medline, PsycInfo, The Cochrane Library, and CINAHL. Articles were eligible if they assessed QOL of opioid-dependent populations using a validated QOL instrument. Item content relevance to opioid-dependent people was evaluated by means of content analysis, and instrument properties were assessed using minimum standards for patient-reported outcome measures.

**Results:**

Eighty-nine articles were retrieved, yielding sixteen QOL instruments, of which ten were assessed in this review. Of the ten instruments, six were disease specific, but none for opioid dependence. Two instruments had good item content relevance. The conceptual and measurement model were described in seven instruments. Four instruments were developed with input from the respective target population. Eight instruments had low respondent and administrator burden. Psychometric properties were either not assessed in opioid-dependent populations or were inconclusive or moderate.

**Conclusions:**

No instrument scored perfectly on both the content and properties. The limited suitability of instruments for opioid-dependent people hinders accurate and sensitive measurement of QOL in this population. Future research is in need of an opioid dependence-specific QOL instrument to measure the true impact of the disease on people’s lives and to evaluate treatment-related services.

**Electronic supplementary material:**

The online version of this article (doi:10.1007/s11136-017-1674-6) contains supplementary material, which is available to authorized users.

## Introduction

Opioid dependence is a chronic relapsing disorder with the greatest disease burden of all illicit drugs [1] and the highest demand for treatment [2]. People dependent on opioids not only suffer from adverse health outcomes and high rates of overdose and overdose deaths [3, 4], but they also often experience negative socioeconomic consequences, social marginalization, and serious long-term impairments in nearly every realm of their lives [5, 6]. Harm reduction programs, such as opioid substitution treatment (OST), are therefore important strategies to reduce the harm of unsafe drug use and the detrimental consequences of drug dependence [7]. OST is a pharmacological treatment preferably administered in combination with psychosocial support, which aims to stabilize patients’ health and enhance their wellbeing [7]. However, the focus in research and clinical practice tends to be on socially desirable outcomes, such as reduced drug use, as opposed to outcomes that are important to the patients themselves, such as their personal wellbeing. Quality of life (QOL) lends itself as a useful parameter to measure the impact of opioid dependence on daily life and to evaluate the quality and success of treatment and harm reduction programs, based on patients’ subjective experiences [8, 9].

QOL refers to an individual’s perception of their position in life in relation to their goals, expectations, standards, and concerns [10]. While patient-reported outcomes (PROs), such as QOL, are widely recognized as valuable outcome measures in treatment [11, 12], the field of opioid dependence is lagging behind in this regard. PROs are outcomes directly reported by the patients themselves, in contrast to clinical outcomes or clinician- or proxy-reported outcomes. In opioid dependence, objective outcomes, such as abstinence from opioids and the reduction of other drug use are generally considered primary measures of treatment success [13]. Yet few opioid-dependent patients achieve sustained abstinence [14] and continued drug use is not necessarily an indicator of poor QOL [15]. Moreover, it is impaired QOL that seems to instigate treatment uptake rather than a desire to reduce drug use per se [16], and research shows that enhanced QOL may sustain remission [17, 18]. Taken together, this underlines the importance of including QOL as a complementary treatment outcome.

The multidimensional concept of QOL is distinct from health-related quality of life (HRQOL). While HRQOL includes physical, psychological, and social domains of health [19], QOL also encompasses life domains beyond health [10]. Given the wide-ranging impact of opioid dependence on people’s lives [6, 20], the concept of HRQOL is limited for use in drug users, providing only a unilateral perspective of their wellbeing [21]. Researchers have been advocating the use of QOL as opposed to HRQOL measures in opioid dependence research, stressing the need for a holistic and integrative approach to treatment [16, 22, 23].

Over the past two decades, there has been increasing research on QOL in opioid-dependent people. A systematic review demonstrated that until 2009, fifteen HRQOL and QOL instruments had been used in 38 studies on people with opioid dependence and about half of the studies used HRQOL rather than QOL instruments [23]. The heterogeneity of tools hampers our ability to compare study outcomes, and the use of HRQOL tools provides a limited view on drug users’ QOL. (Here, *tool* is synonymous with *instrument.*) A further methodological concern that limits our ability to generalize study findings is the use of different types of instruments. QOL instruments can be generic or disease specific. Generic tools allow for comparisons across populations, while disease-specific tools measure aspects that are relevant to a specific population. Instruments can also be uni- or multidimensional. Unidimensional measures provide a global assessment of QOL (e.g., ‘How satisfied are you with your life as a whole?’) whereas multidimensional measures assess satisfaction with multiple life domains.

While research shows that the QOL of opioid users both in and out of treatment is significantly lower compared to the general population [24–26], it is unclear to what extent QOL instruments capture aspects of QOL that are relevant to people with opioid dependence, and whether they are valid and reliable measures for this population. An evaluation of tools is now of utmost importance, because the continued employment of heterogeneous, possibly ill-suited QOL instruments may affect the interpretability and comparability of study outcomes and hinder further advancements in the field [9].

This comprehensive systematic review examines the suitability and quality of QOL instruments for use in people with opioid dependence. The scope of this review is limited to illicit opioid dependence (including patients in substance use treatment, such as OST) and does not include prescription opioid dependence (e.g., chronic pain), as we focus on the context of illegal drug use. We identify QOL instruments that have been used in opioid-dependent populations to date and evaluate the item content relevance to this population, as well as the instruments’ properties (e.g. conceptual and measurement model, psychometric properties). In line with expert recommendations that the way forward in opioid dependence research is a holistic and multidimensional approach to QOL [16, 23], HRQOL tools and single-item measures are excluded from this review.

## Methods

A systematic review of QOL instruments was conducted using an adaptation of the PRISMA guidelines (Preferred Reporting Items for Systematic Reviews and Meta-analysis [27]). We evaluated the item content relevance of instruments by examining the extent to which items reflect QOL domains that are important to opioid-dependent people (indicating ‘suitability’). We also assessed the properties of instruments using recommended minimum standards that PRO measures must meet to be considered suitable for use in scientific studies (indicating ‘quality’).

### Search strategy and inclusion criteria for articles

A comprehensive literature search was performed on 16 March 2017 in the databases MEDLINE (OVID), PsycINFO, The Cochrane Library, and CINAHL (EBSCO). The search strategy included four categories of keywords: (i) quality of life, (ii) instrument, (iii) drug addiction, and (iv) opioids (see Online Resource 1). Reference lists of relevant articles and reviews were screened, a manual Internet search was performed, and colleagues were consulted to identify additional literature and instruments. Authors of articles that could not be accessed were contacted for missing information.

Inclusion criteria for articles were as follows: (1) The QOL of individuals with opioid dependence was assessed. Studies about people with other substance use problems or chronic diseases were included if opioid dependence was present among a subsample of the study *and* if a QOL outcome was reported for that opioid-dependent sample, or for a mixed drug user sample if at least half was opioid dependent. (2) A validated QOL instrument was used. (3) QOL was self-reported by opioid-dependent individuals. (4) Articles were published between 1990 and 2017. This time limit was set, because QOL research in opioid dependence only began in the 1990s. No language restrictions were applied to the search. All identified references were independently reviewed by two of the authors (LS, AM).

### Assessment of item content relevance

Instrument items need to be comprehensive and measure important aspects of a target population’s QOL [28]. We therefore assessed the extent to which instrument items measure QOL domains that have been found relevant to opioid-dependent populations (i.e., a kind of face validity assessment). We used the QOL model of Schalock [29, 30], which was developed for people with intellectual disabilities, but has also been found relevant to opioid-dependent individuals [6, 20], people with mental health problems [31], and other social service recipients [32]. Schalock adopted a sociopolitical perspective, defining quality of life as the promotion of equal opportunities for people with different needs [30]. This makes the model especially useful for studying marginalized populations. The theoretical framework was derived from an extensive review of the QOL literature and consists of eight core domains: emotional wellbeing, interpersonal relations, physical wellbeing, material wellbeing, personal development, self-determination, social inclusion, and rights. While the former four domains are common among models of QOL, it is the latter four that distinguish Schalock’s model. These domains relate to issues of autonomy, social exclusion, and discrimination, which are more pertinent to marginalized populations than to the general population [6, 20]. We propose that if an instrument is to adequately and comprehensively assess the QOL of opioid-dependent individuals, each of Schalock’s QOL domains should be represented by at least one item.

The content of QOL instruments was systematically differentiated by content analysis (as seen in a study by Van Soest-Poortvliet [33]). Four researchers (BS, AB, AM, LS) independently coded the instrument items using MAXQDA software for qualitative data analysis. Each item was assigned to one of Schalock’s eight QOL domains or an additional code ‘global quality of life.’ The latter code was added because instruments often include items that measure QOL on a global scale. Differences in codings were discussed, iteratively, until consensus was reached. Decision rules that were developed during the discussions include:

- Code for meaning, rather than the exact words
- When an item asks about ‘satisfaction with X,’ code X rather than emotional wellbeing (‘satisfaction’)
- When items are subdivided by domains in the original instrument, do not automatically code items as the given domain
- When the instrument instructions say to consider an item in a certain context, also consider that context when coding
- Code ‘global quality of life’ when an item refers to life as a whole or when it can be understood in terms of any of the eight domains

Finally, we compared our codings to the original instrument domains reported in the instrument development papers (see Online Resource 2). This comparison loosely served as a measure of external validity, based on the premise that the distribution of codings should not differ excessively from the original domains.

### Assessment of instrument properties

We assessed the instrument properties using recommended minimum standards for PRO measures developed by the International Society of Quality of Life (ISOQOL). Experienced members of the ISOQOL identified minimum standards for the design and selection of PRO measures that instruments must meet to be considered suitable for use in scientific studies [34]. These recommendations are near identical to the suggested guidelines of the Scientific Advisory Committee of the Medical Outcomes Trust from 15 years ago, underlining the importance and timelessness of these properties [12]. Given that we evaluate the instruments’ suitability for use in opioid-dependent people, we reviewed the psychometric properties when instruments were used in opioid-dependent populations. In addition to the minimum standards, we examined the methodology used to develop the instruments. Methodological rigor is an important aspect in the development of a good PRO measure and yet it appears that few instruments follow systematic ‘gold standard’ development procedures [35].

Five properties were examined at a descriptive level **(**target population, mode of administration, number of items and domains, completion time, availability of languages**)** and six properties were evaluated using assessment criteria (conceptual and measurement model, instrument development methodology, interpretability of scores, responsiveness, reliability, validity). A description of the eleven properties as operationalized in this systematic review is presented in Table 1. Assessment criteria were concretely defined to enhance the inter-rater reliability (Table 2).

**Table 1:** ISOQOL’s recommended minimum standards for PRO measures as operationalized in this review

Properties	Description
1. Target population	A QOL instrument should describe the population it is intended for
2. Conceptual and measurement model	A QOL instrument should describe (i) the conceptual model including how the authors define the concept of QOL or the theoretical framework within which the tool is developed, and (ii) the measurement model including evidence for the dimensionality of the measure
3. Instrument development methodology	Items should be generated with patient input and instruments should be piloted tested [48, 49]
4–6. Mode of administration, number of items and domains, and completion time	A QOL instrument should have low respondent and administrator burden. We examine the mode of administration, the number of items and domains, and completion time
7. Interpretability of scores	The scores of a QOL instrument should be easy to interpret: (i) there should be information on what high and low scores represent, and (ii) norm values should be available
8. Available languages	We report the availability of instruments in different languages
9–11. Responsiveness; Reliability; Construct and content validity	Instruments should have evidence of good responsiveness, internal consistency reliability, construct validity (e.g., convergent and discriminant validity), and content validity in the target population of the research application (here: opioid-dependent people)

**Table 2:** Assessment criteria for six properties based on ISOQOL’s recommended minimum standards for PRO measures

Properties	Score	Assessment criteria
Conceptual and measurement model	2	The conceptual model is described AND evidence is available on the instrument’s dimensionality
	1	The conceptual model is described OR evidence is available on the instrument’s dimensionality
	0	No information found
Instrument development methodology	2	The target population was involved in the item generation AND the instrument was pilot tested
	1	The target population was involved in the item generation OR the instrument was pilot tested
	0	No information found
Interpretability of scores	2	Information on what high and low scores represent AND normative values are available
	1	Information on what high and low scores represent OR normative values are available
	0	No information found
Responsiveness^b^	2	At least moderate effect sizes: SRM^a^ ES ≥ 0.50; Cohen’s *d* ≥ 0.50; Glass’ Δ ≥ 0.50
	1	Below moderate effect sizes: SRM ES < 0.50; Cohen’s *d* < 0.50; Glass’ Δ < 0.50
	0	No information found
Reliability^b^	2	At or above the minimum acceptable value for internal consistency: Cronbach’s *α* ≥ .70
	1	Below the minimum acceptable value for internal consistency: Cronbach’s *α* < .70
	0	No information found
Validity	2	Construct validity AND content validity is reported
	1	Construct validity OR content validity is reported
	0	No information found

^a^Standardized Response Mean

^b^Responsiveness and reliability: When values are reported for multiple items or domains, at least half of the items or domains must be at or above the minimum acceptable value, to rate the property a ‘2’

A data extraction table was used to compare the instrument properties. Two investigators (AM, LS) extracted the relevant information independently in duplicate. Most information was extracted from the instrument development and validation papers, and the instrument manuals. Psychometric properties for opioid-dependent populations were extracted from the 94 studies identified in our literature search. Translations and norm values were found by carrying out an additional search of the literature. Three authors (AB, AM, LS) independently rated the properties and disagreements were discussed until consensus was reached.

## Results

### Identified instruments

**Fig. 1:**
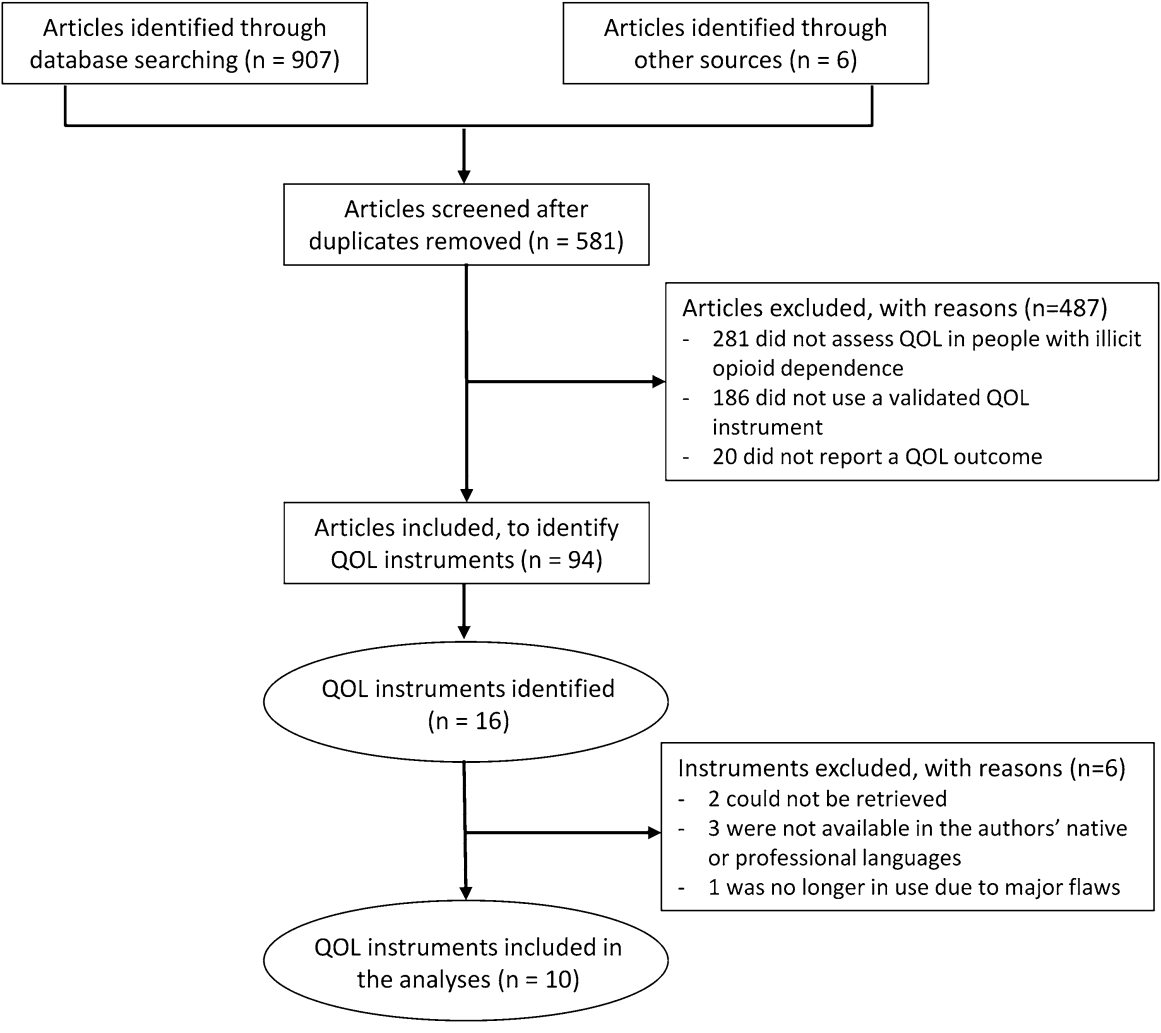
Flow diagram of the selection procedure of articles and instruments

In total, 581 articles were retrieved. Of those, 487 were excluded because they did not assess QOL among people dependent on illicit opioids (*n* = 281), they used HRQOL tools, non-validated QOL tools, or single-item QOL tools (*n* = 186), or they did not report a QOL outcome for an opioid-dependent sample (*n* = 20). Thus, 94 articles used instruments to assess self-reported QOL among opioid-dependent individuals (Fig. 1).

Among the 94 articles, we found 22 differently named instruments, which could be grouped into 16 distinct instruments (Table 3). Measuring instruments were grouped if their content was the same, such as translations, adaptations with a few different items, or different temporal forms of items. WHOQOL-BREF was the most frequently used tool (57 articles), followed by LQoLP-modified in 10 articles, IDUQOL, SWLS, and PWI in 4 articles each, and 11 other instruments in 1–3 articles each. The instruments were developed between 1977 and 2003, ten in the English language and six in a foreign language (LQoLP-modified in Dutch, MSQOL in German, SQLP and TEAQV in French, EBP in Spanish, QOL-DA in Chinese). Thirty-seven articles (39%) were based on studies conducted in Asia (14 in Taiwan, 7 in China, 7 in Malaysia, 6 in India, 3 in Vietnam), 35 articles (37%) were from Europe, 8 articles from Australia, 6 articles from the United States and Canada, 7 articles from the Middle East, and one article was based on a study conducted in multiple countries around the world.

**Table 3:** QOL instruments (*n* = 16) used in studies on opioid-dependent people

Instrument abbreviation (number of articles the instrument was used in)	Instrument and reference
1. WHOQOL-BREF (*n* = 57)^a^	World Health Organization Quality of Life Assessment-Brief Version [10]
2. LQoLP-modified (*n* = 10)^b^	Lancashire Quality of Life Profile-modified [50]
3. IDUQOL (*n* = 4)^c^	Injection Drug User Quality of Life Scale [22, 41]^e^
4. SWLS (*n* = 4)^d^	Satisfaction With Life Scale [51]
5. PWI (*n* = 4)	Personal Wellbeing Index [52]
6. MSQOL (*n* = 3)	Modular System for Quality of Life [53]^e^
7. Q-LES-Q (*n* = 3)*	Quality of Life Enjoyment and Satisfaction Questionnaire [54]
8. SQLP (*n* = 2)*	Subjective Quality of Life Profile [55]
9. TEAQV (*n* = 2)*	Tableau d’Evaluation Assistée de la Qualité de Vie [56]
10. QOLI (*n* = 1)	Quality of Life Inventory [57, 58]^e^
11. QOLI-BV (*n* = 1)	Quality of Life Interview-Brief Version [59, 60]
12. MQOL (*n* = 1)	McGill Quality of Life Questionnaire [61, 62]^e^
13. QLQ (*n* = 1)	Quality of Life Questionnaire [63]
14. ComQoL-A5 (*n* = 1)*	Comprehensive Quality of Life Scale—Adult [64]
15. EBP (*n* = 1)*	Escala Bienestar Personal [65]
16. QOL-DA (*n* = 1)*	Quality of Life Measurement for Drug Addicts [66]

Note that two articles used two QOL instruments each [67, 68]

^a^Similar versions grouped under WHOQOL-BREF: (i) WHOQOL-BREF (*n* = 39) was used in eleven languages: Malay, Chinese, Hindi, Persian, Vietnamese, Slovenian, Slovak, Spanish, German, English, Italian; (ii) WHOQOL-BREF-Taiwanese version (WHOQOL-BREF-TW, *n* = 13)

^b^Similar versions grouped under LQoLP-modified: (i) Lancashire Quality of Life Profile-modified (LQoLP-modified, *n* = 3), (ii) Berlin Quality of Life Profile (Berliner Lebensqualitätsprofil, BELP, *n* = 6), (iii) Manchester Short Assessment of Quality of Life (MANSA, *n* = 1)

^c^Similar versions grouped under IDUQOL: (i) Injection Drug User Quality of Life Scale (IDUQOL, *n* = 3), (ii) Drug User Quality of Life Scale (DUQOL, *n* = 1)

^d^Similar versions grouped under SWLS: (i) Satisfaction With Life Scale (SWLS, *n* = 2), (ii) Temporal Satisfaction With Life Scale (TSWLS, *n* = 1), (iii) Temporal Satisfaction With Life Scale - present (TSWLS-present, *n* = 1)

^e^The tools examined in this review are taken from the instrument development articles referenced in Table 1, except if the most frequently used version of an instrument differed from the original. We examined (i) the 21-item IDUQOL from Hubley and colleagues [41], instead of the 17-item version from Brogly and colleagues [22], (ii) the 16-item QOLI from Frisch [58], instead of the earlier 17-item version from Frisch and colleagues [57], (iii) the 59-item MSQOL that we received from the author, instead of the version from Pukrop and colleagues [53], and (iv) the 16-item MQOL from Cohen and colleagues [62], instead of the earlier 17-item version from Cohen and colleagues [61]

* Instruments marked with an asterix are excluded from further analyses

Of the sixteen instruments, six were excluded from analysis, because they could not be retrieved (SQLP, Q-LES-Q), they were not available in the authors’ native or professional languages English, German, or Dutch (TEAQV in French, EBP in Spanish, QOL-DA in Chinese), or the instrument had already been abandoned by the author due to major flaws (ComQol [36]). A total of ten instruments were included in the analysis. We assessed the most frequently used version of each instrument, which was incidentally also the most recently developed version.

### Item content

**Fig. 2:**
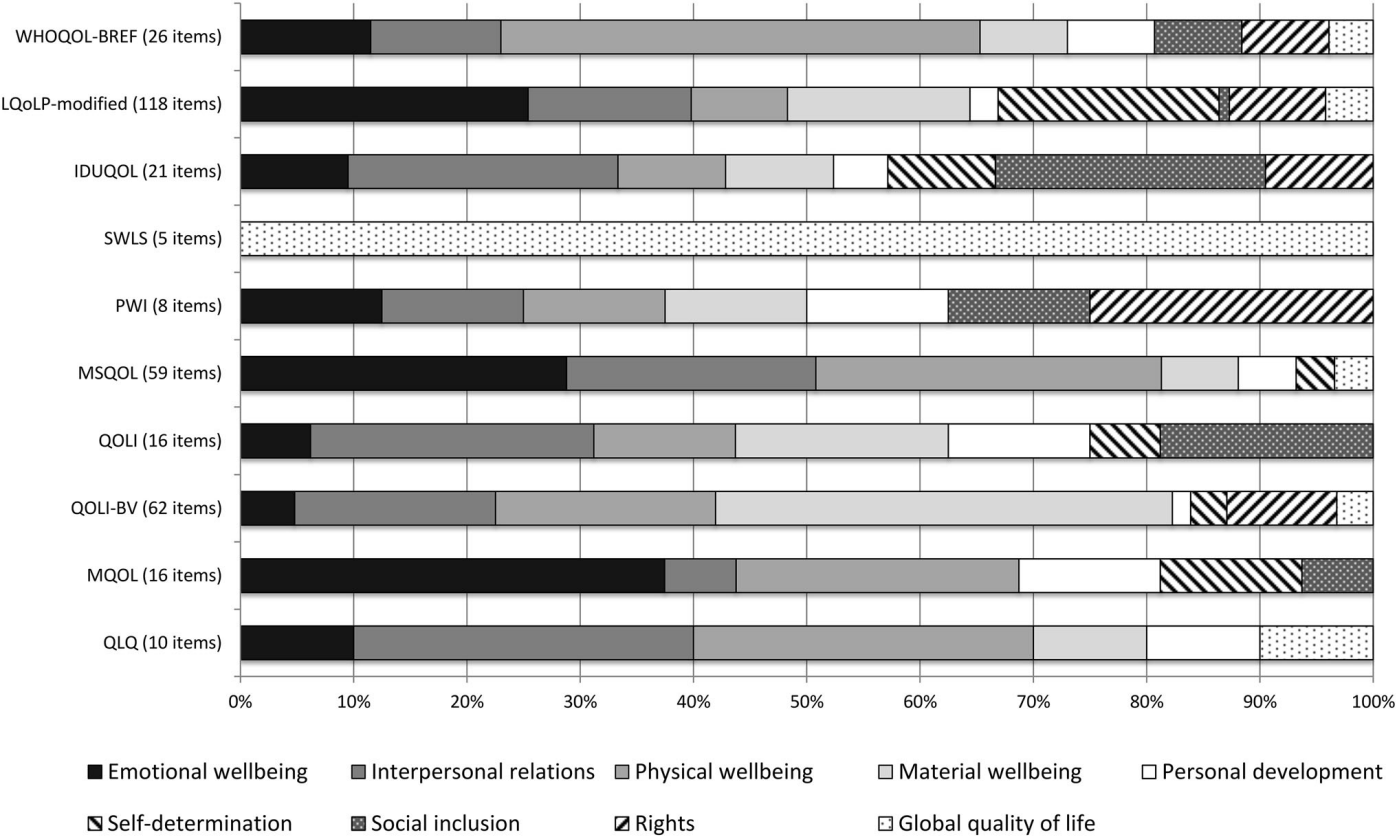
Schalock’s eight quality of life domains captured by the 10 instruments

The 341 items of the 10 instruments were assigned to Schalock’s eight QOL domains or a global QOL category. Four independent coders initially agreed on 71% of the items. Coding agreement was highest for the domain’s emotional wellbeing, physical wellbeing, interpersonal relations and material wellbeing. Figure 2 presents information on the QOL domains captured by each instrument. Overall, the domain’s emotional wellbeing, interpersonal relations, physical wellbeing, and material wellbeing were coded more frequently across instruments than the other four domains. Global QOL items were found in six instruments. Only two instruments, LQoLP-modified and IDUQOL, include at least one item on each of Schalock’s eight domains. The five least-frequently used tools (MSQOL, QOLI, QOLI-BV, MQOL, QLQ) have no items on social inclusion and/or rights. WHOQOL-BREF, PWI, and QLQ do not assess self-determination.

Instruments vary greatly in their item content focusing on different QOL domains. WHOQOL-BREF comprised 42.3% and MSQOL comprised 30.5% physical wellbeing items, followed by emotional wellbeing (WHOQOL-BREF 11.5%; MSQOL 28.8%) and interpersonal relations (WHOQOL-BREF 11.5%; MSQOL 22.0%). Both instruments include fewer or no items in the remaining domains.

LQoLP-modified and IDUQOL are the only two instruments that comprised at least one item in each of Schalock’s eight QOL domains. LQoLP-modified focuses more on emotional wellbeing (25.4%), interpersonal relations (14.4%), material wellbeing (16.1%), and self-determination (19.5%), and has only 1 out of 118 items relating to social inclusion. IDUQOL focuses on the social domains (interpersonal relations 23.8%; social inclusion 23.8%), while items are evenly distributed for the remaining domains (4.8–9.5%).

SWLS measures exclusively global QOL. QOLI has 1–4 items on each domain except rights, and measures more interpersonal relations, social inclusion, and material wellbeing (3–4 items) than the other domains (1–2 items). QOLI-BV has a large percentage of items pertaining to material wellbeing (40.3%), followed by physical wellbeing (19.4%), interpersonal relations (17.7%), and up to 9.7% on the remaining domains. MQOL measures emotional wellbeing (37.5%), followed by physical wellbeing (25.0%), and 0 to 2 items on the remaining domains.

The domain distribution of PWI and QLQ must be interpreted with caution due to the small number of items (8 and 10, respectively). PWI items are evenly distributed across domains with about 1 item per domain. QLQ includes 1–3 items per domain, with a greater focus on interpersonal relations and physical wellbeing, and no items on self-determination, social inclusion, and rights.

As a measure of external validity, our codings were compared to the original instrument domains. We found that the original domains were mostly reflected in our codings. For instance, over 80% of MSQOL items were coded emotional wellbeing, physical wellbeing, or interpersonal relations. Similarly, most of MSQOL’s original domains pertained to those life domains. However, there were also differences to the original domains. Over 40% of WHOQOL-BREF items were coded physical wellbeing, which differs from the original WHOQOL-BREF domains, in which ‘physical health’ is one of four domains (i.e., 25%). This suggests that WHOQOL-BREF has a greater focus on health than indicated by the original domains.

### Instrument properties

Characteristics of the ten QOL instruments are presented in Table 4. Four generic tools (WHOQOL-BREF, SWLS, PWI, QOLI) and six disease-specific tools were identified, of which four are for mental health care populations (LQoLP-modified, MSQOL, QOLI-BV, QLQ), one for people with life-threatening or terminal illness (MQOL), and one for injection drug users (IDUQOL). Seven tools are in questionnaire format and three are applied as structured interviews (LQoLP-modified, IDUQOL, QOLI-BV). Most tools contain 5–26 items measuring QOL, whereas MSQOL, QOLI-BV, and LQoLP-modified contain 48, 74, and 133 QOL items, respectively. Thus, two of the three tools in interview format are also the longest tools (LQoLP-modified and QOLI-BV).

**Table 4:** Description of QOL instruments (*n* = 10) used in opioid-dependent people according to ISOQOL’s recommended minimum standards for PRO measures

Instrument	Target population	Mode of administration	Number of items and domains	Completion time (minutes)	(Number of) Available languages	Conceptual and measurement model	Instrument development methodology	Interpretability of scores	Responsiveness	Reliability	Construct and content validity
1. WHOQOL-BREF	People in need of health care; any clinical group	Questionnaire	26 items, 4 domains	NR^a^	English + 40^+^	2	2	2	1–2^b^	2^b^	1^b^
2. LQoLP-modified	People with mental illness; patients in mental health care settings	Structured interview	133 items^g^, 10 domains	45	Dutch, Flemish	2	2	1	0	0	0
3. IDUQOL	Injection drug users	Structured interview using titled picture cards depicting life areas and a Likert scale	21 items, 21 domains	20	English, French, Spanish	2	2	1	0	2^c^	1^c^
4. SWLS	General population	Questionnaire	5 items	NR	English + 32	2	0	2	0	0	0
5. PWI	General adult population	Questionnaire	7 items + 1 optional item, 7 domains + 1 optional domain	NR	English + 27	2	1	2	0	2^d^	0
6. MSQOL	Psychiatric population	Questionnaire	Core module: 48 items, 6 domains + Optional modules: 11 items, 4 domains	NR^h^	English + German	1	0	1	0	2^e^	1^e^
7. QOLI	For people with mental disorders or physical illnesses, or to assess community-wide social problems	Questionnaire	16 items, 16 life areas	10	English	1	0	2	0	0	0
8. QOLI-BV	People with severe mental illness	Structured interview	74 items^g^, 8 domains	16	English, French, Spanish, Danish, Indian	2	1	1	0	2^f^	1^f^
9. MQOL	People with life-threatening or terminal illness	Questionnaire	16 items, 5 subscales	15-35	English + 15	2	1	1	0	0	0
10. QLQ	People in psychotherapy	Questionnaire	10 items	NR	English	0	2	1	0	0	0

*NR* not reported in the instrument development and validation papers or manuals

^a^Skevington and McCrate [69] reported completion time subsample means for the WHOQOL-BREF, ranging from 4.5 min (students) to 20 min (rehabilitation)

^b^Five studies assessed the psychometric properties of WHOQOL-BREF in opioid-dependent populations [70–74]

^c^Castillo [75] reported psychometric properties for IDUQOL

^d^Miller and colleagues [76] reported psychometric properties for PWI

^e^Karow and colleagues [26] reported psychometric properties for MSQOL

^f^Wasserman and colleagues [77] reported psychometric properties for QOLI-BV

^g^Reported item number differs from the number of items assessed in the content analysis. LQoLP-modified includes items for the administrator and items on domain importance, leaving 118 QOL items for content analysis. QOLI-BV includes questions on Sociodemographics, leaving 62 QOL items for content analysis

^h^Pukrop reported in an unpublished manuscript that completion time was 7–30 min depending on the state of the psychiatric patient

Completion time was only reported for half the tools and ranged from 10–35 min (IDUQOL, QOLI, QOLI-BV, MQOL) and 45 min for LQoLP-modified. Four tools are available in more than 16 languages (WHOQOL-BREF, SWLS, PWI, MQOL), five tools in English and up to 4 other languages (IDUQOL, MSQOL, QOLI, QOLI-BV, QLQ), and LQoLP-modified is available in Dutch and Flemish.

The conceptual and measurement model are described in nearly all tools, except MSQOL, QOLI, and QLQ, which lack a description of the conceptual model or evidence for the dimensionality, or both. Instrument development methodologies vary greatly between tools. For the three most frequently used instruments (WHOQOL-BREF, LQoLP-modified, IDUQOL) and QLQ, items were generated with input from the target population and the instrument was piloted. For PWI, QOLI-BV, and MQOL, items were generated with target population input *or* they were piloted. The remaining tools were developed from already-existing instruments or a review of the literature, and they were not piloted. The interpretability of outcomes is high in WHOQOL-BREF, SWLS, PWI, and QOLI, with information on what high and low scores represent and available normative values, while the other six tools lack normative values.

Data on psychometric properties for opioid-dependent populations were scarce and incomplete. Psychometrics of five instruments (WHOQOL-BREF, IDUQOL, PWI, MSQOL, QOLI-BV) was tested in nine studies, of which five were on WHOQOL-BREF. *Responsiveness* was only assessed for WHOQOL-BREF and yielded mixed evidence, with one study demonstrating above moderate effect sizes (Cohen’s *d* ≥ 0.50 for all four WHOQOL-BREF domains) and another study failing to reveal any significant changes over time (below moderate effect sizes: Cohen’s *d* < .50 for all four domains). *Reliability* was above the minimum acceptable value for internal consistency for five instruments (all Cronbach’s *α* ≥ .70); no information on reliability was reported for the other five instruments. *Content validity* was not tested in any tool. *Construct validity* was assessed in four instruments (WHOQOL-BREF, IDUQOL, MSQOL, QOLI-BV) although evidence varies. Three studies demonstrated acceptable or marginal goodness-of-fit of WHOQOL-BREF (via Rasch Analysis or Confirmatory Factor Analysis), but with misfit items, new emerging domains, or only a good fit after making adjustments to the questionnaire. One study demonstrated adequate fit indices for IDUQOL (GFI = .92; CFI = .97; RMSEA = .044). Adequate convergent and discriminant validity was demonstrated for MSQOL (moderate and low correlations with a range of variables), and evidence of convergent and discriminant validity of QOLI-BV was suggested by moderately high and low correlations, respectively.

Finally, we examined whether instruments include subjective and/or objective items. While QOL comprises both subjective and objective components, the subjective component prevails and research tends to focus increasingly on QOL as a subjective concept [30]. We found that seven instruments assess exclusively subjective QOL, while three instruments assess both subjective and objective QOL. LQoLP-modified and MSQOL consist about one-third and QOLI-BV more than half of objective items. Objective items include “Do you have a paid job?” (LQoLP-modified) and “What is your current living situation?” (QOLI-BV), whereas subjective items include “How satisfied are you with your income?” (LQoLP-modified) and “How do you feel about the living arrangements where you life?” (QOLI-BV).

## Discussion

Despite increasing use of QOL measures in studies on opioid-dependent people, no suitable QOL instrument is available to date. When selecting an instrument, both its quality and content are important considerations. Yet, no instrument in this review scored perfectly on the recommended minimum standards for PRO measures *and* comprehensively assessed QOL according to Schalock’s model [29, 30]. Only IDUQOL and LQoLP-modified had good item content relevance. However, their high respondent and administrator burden (interview format, long completion time) make them less practical for repeated use in research and routine patient care. Moreover, they had little evidence of good psychometric properties in opioid users. On the other hand, WHOQOL-BREF was the only tool that scored adequately on the properties, but its item content focuses on physical health, thereby providing a limited view of drug users’ QOL. This is a critical observation because the majority of studies in this review used WHOQOL-BREF. Other instruments have further limitations. For instance, SWLS is a measure of global QOL and so does not provide a multidimensional assessment of QOL, and QOLI-BV assesses largely material wellbeing, thus providing limited insight into individuals’ overall QOL.

The strength of this review lies in its structured approach to the instrument evaluation using a theoretical framework and well-established standards for PRO measures. Results highlight limitations in item content and properties that need to be addressed in QOL instruments in the future. Particularly evidence of validity, reliability, and responsiveness in opioid-dependent populations was scarce across tools. The field of QOL measurement in opioid dependence is in its infancy. This becomes particularly evident when comparing the results to other instrument reviews. Many medical disciplines have multiple disease-specific instruments available for any one condition, and reviews determine the ‘best’ instrument by assessing psychometric properties using detailed quality criteria [37]. This would not have been possible in this review considering the lack of disease-specific tools and scarce evidence of psychometric properties. Additionally, instruments in this review scored rather poorly on the properties. A reason for this might be that most tools were developed before the year 2000, while numerous guidelines and criteria for the transparent development and psychometric evaluation of PRO measures were developed in more recent years [37–39]. We strongly recommend that available guidelines and criteria be applied in the development of new QOL instruments.

Item content relevance of an instrument for a given study population is important and must be investigated if the population differs from the one in which the tool was developed [38]. Only IDUQOL was developed for drug users and no tools specifically for opioid-dependent people. Accordingly, IDUQOL was the only one of two tools that comprehensively measured Schalock’s domains. The other instruments were developed for (and with input from) the general population or broad mental health populations. Overall, the instruments varied greatly in their content, focusing on different life domains. The different foci may in part be due to the lack of a universally accepted definition of QOL and because researchers operationalize the concept differently. Researchers need to be aware of the different conceptualizations of QOL when selecting an instrument for a study, as the item content needs to match the study population. It should be noted that, in the current literature, self-determination is no longer always seen as a domain of QOL (as in Schalock’s model) but rather a pre-requisite of QOL. The self-determination theory [40] proposes that individuals’ wellbeing is determined by the fulfillment of three basic needs: autonomy, competence, and relatedness. This could change how we approach the concept of QOL in future research.

The field of opioid dependence is in need of a high-quality, disease-specific QOL instrument [9, 22, 23, 41, 42]. While research consistently shows that the QOL of opioid-dependent populations is poor, the use of HRQOL and generic tools may undermine our understanding of the extent and severity of the impact of the disease, as well as overestimate the effectiveness of treatment. The advantage of disease-specific instruments is that they provide more relevant and sensitive results than generic instruments, which are applicable across populations [43]. Qualitative research revealed that specific barriers in the domains of social inclusion, rights, and self-determination reduce the QOL of people with opioid dependence [6, 20]. However, it is especially these QOL domains that were underrepresented in the instruments in this review. In order to develop a valid and reliable QOL instrument, more qualitative research will be essential to identify what is most important to opioid-dependent people for a good QOL and what needs to be included in an instrument.

Another important observation regarding the literature is that researchers often did not use the original, validated instrument but a variation of the tool. Researchers added or removed items seemingly at random (e.g., SWLS, MSQOL) or developed a number of different but very similar versions of an instrument (e.g., LQoLP-modified, IDUQOL). We speculate that researchers wanted to adapt the instruments to meet the study needs. However, this hinders the assessment and comparison of outcomes. Moreover, sixteen different tools were used in 94 articles. Increased uniformity of instruments used would enhance the interpretability of results and the comparability of outcomes across studies. We also found that about half of the articles used HRQOL as opposed to QOL instruments, meaning that measures of HRQOL were still used as much as nearly a decade ago [23]. While HRQOL instruments are useful to gain insight on the impact of a disease on patients’ functioning, researchers need to be aware that the term HRQOL refers to patients’ self-perceived health status and is not synonymous to QOL [44, 45].

Limitations to this review relate to assumptions we made and possible sources of bias. First, we assumed that Schalock’s domains are key components to a good QOL for opioid-dependent individuals. Schalock’s model has not been extensively tested in opioid users yet. Nevertheless we chose this model because the eight domains have been found to be pertinent to opioid users, as well as broader groups of drug users and other marginalized populations [6, 46, 47]. Second, the coding of instrument items involved a somewhat subjective evaluation. We tried to reduce the subjectivity by engaging four independent coders, developing coding rules, and discussing disagreements in a consensus meeting. Third, our selection of instruments may be biased. Five instruments were excluded from analysis because they could not be retrieved or were not available in the author’s languages. However, we did assess the most frequently used tools, which are arguably more relevant to the literature. Also the frequency of instrument use may be biased. Foreign language instruments are more popular in their respective countries and used more in local journals, which we did not target in our literature search. Finally, it should be noted that the results of the content analysis do not indicate opioid dependence specificity of instruments. This would require an assessment of content validity. But seeing as the QOL of opioid-dependent people is not precisely defined yet, we took a more conservative approach and assessed broad QOL domains that have been shown to be pertinent to opioid users [6, 20]. A next step might be to assess the content validity of instruments that performed well in our content analysis.

Opioid-dependent people make up the largest proportion of patients seeking drug treatment and they suffer wide-ranging detrimental impacts on their QOL. Yet no suitable instrument is available to measure QOL in this population. This review enables researchers to make an informed decision when selecting a QOL tool, and it enables improved interpretation of the literature (e.g., by knowing that certain instruments measure largely health-related aspects of QOL). Furthermore, this systematic review highlights the pressing need of a multidimensional QOL instrument that is specific to opioid-dependent populations. The development of such tool is critical for advancements in the field. A disease-specific tool will provide more relevant and valid data and thereby more accurate assessment of the impact of the disease and treatment on people’s QOL. Moreover, it will demonstrate patient needs, providing an incentive for improving treatment and patient-centered drug policy. We especially recommend the development of a short QOL questionnaire that is practical to use in routine patient care, in order to further bridge the gap between research and practice.

## Electronic supplementary material

Below is the link to the electronic supplementary material.

Supplementary material 1 (DOCX 52 kb)
