# Supplementary material for: Measuring quality of life in opioid-dependent people: a systematic review of assessment instruments
Source: Qual Life Res. 2017 Jul 31;26(12):3187–200. doi: 10.1007/s11136-017-1674-6 (PMC5681984; doi:10.1007/s11136-017-1674-6)
Supplement: Supplementary file 1 — Supplementary material 1 (DOCX 52 kb) [file 11136_2017_1674_MOESM1_ESM.docx]

**Article title:** Measuring quality of life in opioid dependent people: A systematic review of assessment instruments

**Journal:** Quality of Life Research

**Author names:** Lisa Strada, Wouter Vanderplasschen, Angela Buchholz, Bernd Schulte, Ashley E. Muller, Uwe Verthein, Jens Reimer

**Corresponding author:**

Lisa Strada

Centre for Interdisciplinary Addiction Research, University Medical Centre Hamburg-Eppendorf, Martinistrasse 52, 20246 Hamburg, Germany

E-email address: L.strada@uke.de

**Supplementary material:** See below

**Online Resource 1**

Full electronic search strategy for MEDLINE database

Database(s): Ovid MEDLINE(R) 1946 to March Week 2 2017, Ovid MEDLINE(R) In-Process & Other Non-Indexed Citations March 16, 2017

| **#** | **Searches** | **Results** |
| --- | --- | --- |
| 1 | quality of life.mp. or "Quality of Life"/ | 260013 |
| 2 | ((quality adj2 life) or (satisfaction adj2 life)).ab,ti. | 211249 |
| 3 | ("subjective wellbeing" or "personal wellbeing" or "subjective well-being" or "personal well-being").ab,ti. | 2640 |
| 4 | 1 or 2 or 3 | 267047 |
| 5 | questionnaire.mp. or Questionnaires/ | 536884 |
| 6 | instrument.mp. | 96044 |
| 7 | (instrument* or scale* or questionnaire* or test* or measure* or assess*).ab,ti. | 6539319 |
| 8 | 5 or 6 or 7 | 6628096 |
| 9 | substance related disorders.mp. or Substance-Related Disorders/ | 88150 |
| 10 | substance abuse.mp. | 45867 |
| 11 | Substance Abuse, Intravenous/ | 13798 |
| 12 | Syringes/ | 5690 |
| 13 | Drug Users/ | 2146 |
| 14 | ((addict* or depend* or use* or using or abus*) adj1 (drug* or substance*)).ab,ti. | 144619 |
| 15 | addiction.mp. | 35626 |
| 16 | addiction.ab,ti. | 33406 |
| 17 | 9 or 10 or 11 or 12 or 13 or 14 or 15 or 16 | 220310 |
| 18 | heroin dependence.mp. or Heroin Dependence/ | 8801 |
| 19 | opioid related disorders.mp. or Opioid-Related Disorders/ | 10572 |
| 20 | opiate addiction.mp. | 787 |
| 21 | (opioid* or opiate* or heroin or methadone or buprenorphine or "injecting drug use*" or "injection drug use*").ab,ti. | 108503 |
| 22 | 18 or 19 or 20 or 21 | 112571 |
| 23 | 4 and 8 and 17 and 22 | 402 |
| 24 | limit 23 to yr="1990 -Current" | **399** |

**Online Resource 2**

Original domains of the ten instruments as reported in the instrument development articles

| **WHOQOL-BREF** | **LQoLP- modified** | **IDUQOL** | **SWLS** | **PWI** | **MSQOL** | **QOLI** | **QOLI-BV** | **MQOL** | **QLQ** |
| --- | --- | --- | --- | --- | --- | --- | --- | --- | --- |
| **4 domains** | **10 domains** | **21 domains** | **Not reported** | **8 domains** | **10 domains** | **16 life areas** | **8 domains** | **5 subscales** | **10 life areas** |
| Psychological, | Positive self-esteem, Negative self-esteem, | Feeling good, |  |  | Affect, | Love,  Self-esteem, |  | Psychological, | Loving,  Self-acceptance, |
| Social relationships, | Family relations, | Family, Friends, Partner(s),  Sex, |  | Personal Relationships, | Psychosocial relationships, Family, Partner, Children, | Friends, Children, Relatives, | Family,  Social relations, | Support, | Social contact, Parenting, |
| Physical health, | Health, | Health,  Health care, |  | Personal Health, | Physical health, Vitality, | Health, | Health, | Physical symptoms, Physical wellbeing, | Eating, Sleeping, |
| Environment | Living situation, Finances, | Housing, Education, Money, Transportation, |  | Standard of Living, | Material wellbeing, Occupation, | Home,  Money,  Work, Learning, | Living situation, Finances, Work and school, |  | Working, Earning, Environment, |
|  | Leisure and social participation, Safety, Framework, Fulfillment (and objective items on life domains, e.g. occupation, psychological problems, religion) | Leisure activities, Drugs, Neighborhood Safety,  Drug Treatment,  Harm Reduction,  Independence, Treatment by others, Resources in the community,  Being useful, Spirituality |  | Personal Safety,  Future Security, Community-Connectedness, Achieving in Life, Spirituality/ Religion | Leisure | Play, Creativity, Neighborhood, Community, Helping,  Goals and values | Daily activities and functioning, Legal and safety issues | Existential wellbeing | Leisure |

Note, the domains are roughly presented in the following order: Emotional wellbeing, Interpersonal relations, Physical wellbeing, Material wellbeing, and other.
